# Acceptability of Massage with Skin Barrier-enhancing Emollients in Young Neonates in Bangladesh

**Published:** 2007-06

**Authors:** A.S.M. Nawshad Uddin Ahmed, Samir K. Saha, M.A.K. Azad Chowdhury, Paul A. Law, Robert E. Black, Mathuram Santosham, Gary L. Darmstadt

**Affiliations:** 1 Department of Neonatology, Bangladesh Institute of Child Health, Dhaka Shishu Hospital, Dhaka, Bangladesh; 2 Department of Pediatrics, Kumudini Women's Medical College, Mirzapur, Tangail, Bangladesh; 3 Department of Microbiology, Bangladesh Institute of Child Health, Dhaka Shishu Hospital, Dhaka, Bangladesh; 4 Department of Medical Informatics, Kennedy Krieger Institute, Johns Hopkins Medical Institutions, Baltimore, MD, USA; 5 Department of International Health, Bloomberg School of Public Health, Johns Hopkins University, Baltimore, MD

**Keywords:** Bathing, Emollient, Oil massage, Prospective studies, Skin, Bangladesh

## Abstract

Oil massage of newborns has been practised for generations in the Indian sub-continent; however, oils may vary from potentially beneficial, e.g. sunflower seed oil, to potentially toxic, e.g. mustard oil. The study was carried out to gain insights into oil-massage practices and acceptability of skin barrier-enhancing emollients in young, preterm Bangladeshi neonates. Preterm infants of <33 weeks gestational age were randomized to high-linoleate sunflower seed oil, Aquaphor Original Emollient Ointment™, or the comparison group (usual care). A survey was administered at admission to assess routine skin-care practices prior to admission and at discharge to assess acceptability of emollient therapy during hospitalization. Oil massage was given to 83 (21%) of 405 babies before hospital admission, 86% (71/83) of whom were delivered at home. Application of oil, most commonly mustard oil (88%, 73/83), was started within one hour of birth in 51 cases (61%) and was applied all over the body (89%, 74/83) one to six (mean 2.2) times before admission. Of infants who received emollient therapy in the hospital, 42% (n=32) of mothers reported that the emollient applied in the hospital was better than that available at home, and only 29% would use the same oil (i.e. mustard oil) in the future as used previously at home. No problems resulted from use of emollient in the hospital. Topical therapy with sunflower seed oil or Aquaphor was perceived by many families to be superior to mustard oil. If caregivers and health professionals can be motivated to use inexpensive, available emollients, such as sunflower seed oil that are beneficial, emollient therapy could have substantial public-health benefit.

## INTRODUCTION

Oil massage of newborns has been practised routinely for generations throughout the Indian sub-continent and Mediterranean region (1–7). Traditional oil massage presumably evolved in developing countries in response to perceived benefits. Few reports are available, however, detailing the practice of and perceptions about this treatment, which may be particularly beneficial in preterm infants during the early neonatal period when their skin barrier is most highly compromised (8–12).

While performing a study to evaluate the effect of topical treatment with skin barrier-enhancing emollients on prevention of nosocomial infections in preterm infants (13), we also sought to gain insights into the epidemiology, practice, and perceptions regarding oil massage of young Bangladeshi neonates and to assess the acceptability of modified oil-massage practices, including use of skin barrier-enhancing emollients.

## MATERIALS AND METHODS

### Study site and subjects

This prospective study was conducted in the Special Care Nursery at Dhaka Shishu (Children) Hospital in a cohort of preterm neonates admitted during December 1998-July 2003. The study was part of the trial of the impact of topical emollient therapy on incidence of nosocomial infections reported previously (13). Newborn infants of <33 weeks gestational age and <72 hours old and whose parents or guardians provided informed consent were eligible for enrollment. The Ethical Review Board of Dhaka Shishu (Children) Hospital and the Committee for Human Research at the Johns Hopkins Bloomberg School of Public Health approved the study protocol. The trial was registered at clinicaltrials.gov (no. 98-04-21-03-2).

### Intervention

A study nurse randomly assigned eligible infants to an intervention group—high-linoleate sunflower seed oil (Omega Nutrition, Bellingham, WA, USA) or Aquaphor Original Emollient Ointment™ (Beiersdorf, Norwalk, CT, USA) composed of petrolatum, mineral oil, mineral wax, and lanolin alcohol—or the comparison group (usual care). The nurses applied emollient (sunflower seed oil or Aquaphor^TM^) to the entire body surface, except the scalp and face, of infants assigned to the intervention groups three times daily for the first 14 days and then two times daily until discharge. Infants in the control group received the standard skin care for the Special Care Nursery, which did not include any massage or use of topical emollients.

### Data collection

Baseline characteristics and anthropometric measurements of patients were recorded at enrollment. Information was collected regarding whether any oil or other topical skin-care products was used prior to admission, and if so, its name, purity, age-at-first application, site, frequency and duration of application, reasons for applying the product and whether it caused any problem, and whether the product, particularly if it was an oil, was fed to the baby.

At the time of discharge, families whose infants received emollient therapy in the hospital were asked to comment on the emollient used and whether it caused any problem and on their anticipated oil-massage practices at home in the future.

### Data analysis

The Epi Info software (version 6) (Centers for Disease Control and Prevention, Atlanta, GA) was employed for recording data and validation of dual entries. The SPSS software (version 12.0 for Windows) (SPSS Inc., Chicago, IL, USA) was used for analysis of data. Results were verified by conducting standard tests for significance (p<0.05), including unpaired Student's t-test and chi-square test, as appropriate.

## RESULTS

### Characteristics of patients

In total, 497 patients were enrolled in the emollient trial, and 405 completed the baseline survey on oil-massage practices prior to admission; 281 died, 64 left the hospital against medical advice, and 31 were lost to follow-up for this study. Thus, 121 families participated in the follow-up study of emollient acceptability. Families who completed the skin-care questionnaire were similar in characteristics to the larger group enrolled in the emollient trial and were balanced across treatment groups (data not shown).

### Oil massage before admission

Oil massage was given to 83 (20.5%) of the 405 babies before hospital admission; 71 (85.5%) of 83 babies who were treated with emollient were delivered at home, while 77% of those surveyed were delivered in a hospital or clinic. Of those who received an oil massage, the most commonly-used product was mustard oil, which was applied to 73 (88%) of the 83 babies massaged; other products mentioned were coconut oil in eight (9.6%) cases, and olive oil and proprietary baby lotion in one each. Oil used was pure, except in one case in which garlic was added to mustard oil. Application of oil started from just after birth, and in 51 (61.4%) cases, it was applied within one hour of birth; the mean age at application was 4.4 (SD 8.1) hours. Oil was applied one to six times (mean 2.19, SD 1.30) before admission to the hospital, all over the body in 74 (89.2%) of the 83 babies. Eight babies were fed mustard oil, ranging from one to five mL; one baby was fed oil three times, and the others were fed oil just once before admission.

Oil was applied for various reasons, principally to keep the baby warm (n=37, 22%), prevent infections (n=30, 18%), and improve the condition of the skin (n=10, 6%) or the overall health of the baby (n=14, 8%). No-one complained of any problem relating to the oil applied. The practice of oil massage was significantly more common among births at home and when the mother was uneducated (Table).

**Table:** Infuence of factors on use of oil massage in young neonates prior to admission

Factor	Total	Oil applied		Oil not applied		p value
		No.	%	No.	%	
Residence						
Urban	217	50	64.1	167	55.9	
Suburban	62	6	*7.7*	56	18.7	0.064
Rural	98	22	28.2	76	25.4	
Total	377	78	100	299	100	
Season of birth						
Spring	116	23	27.7	93	28.2	
Summer	115	28	33.7	87	26.4	
Autumn	104	18	21.7	86	26.0	0.567
Winter	78	14	16.9	64	19.4	
Total	413	83	100	330	100	
Place of birth						
Home	116	71	85.5	45	14.0	
Hospital/clinic	289	12	14.5	277	86.0	0.000
Total	405	83	100	322	100	
Maternal education						
Illiterate	37	14	17.5	23	7.2	
Up to Class V	114	30	37.5	84	26.4	
Class VI to XII	208	35	43.8	173	54.4	0.001
≥Class XIII	39	1	1.2	38	12.0	
Total	398	80	100	318	100	
Maternal occupation						
Housewifery	279	34	91.9	245	88.4	
Service	35	3	8.1	32	11.6	0.557
Total	314	37	100	277	100	
Socioeconomic class (total monthly income in Taka)						
<2,000	16	6	9.0	10	3.9	
2,000-3,999	72	20	29.8	52	20.0	0.321
4,000-7,999	134	23	34.3	111	42.9
≥8,000	104	18	26.9	86	33.2	
Total	326	67	100	259	100

### Acceptability of emollient-use in hospital

Emollient was applied in 77 of the 121 babies followed up (sunflower seed oil, n=33; Aquaphor^TM^, n=44; comparison group, n=44). Thirty-two (41.6%) mothers replied that the emollient applied in the hospital was better than what they would have used at home, 10 (13.0%) indicated that they would not have used oil massage at home, and one (1.3%) thought that there was no difference between the oil used in the hospital and the oil they would use at home; 34 (44.1%) did not know.

Results of qualitative research among this group suggested that, while in hospital they came to know that mustard oil may not be good for newborns, but they did not know whether the oil/emollient applied in the hospital would be available in the future. For this reason, when asked about their anticipated emollient-therapy practices in their next baby, 55.4% (n=67) of the 121 mothers indicated that they did not know what they would do, 28.9% would use the same oil as they had used previously at home, and 15.7% definitely would not use the same oil as used at home in the past. The mothers reported no problems with the use of emollient during hospital stay, including no rashes; no relation to vomiting, fever, cough and cold, or any illness; it did not weaken the baby; did not cause the baby to slip from the hands during care; and did not make it more difficult to keep the baby clean.

## DISCUSSION

Oil massage is routinely practised in Bangladesh. Results of a previous study among families with children presenting for care at our institution indicated that more than 96% of caregivers had practised oil massage, irrespective of socioeconomic status, place of residence, and whether the infant had been term or preterm (8). In this study among preterm infants aged less than 72 hours, one in five had already been given oil massage before being admitted to the hospital, irrespective of socioeconomic status and place of residence, education, or occupation of mother. Oil massage was significantly more common among infants delivered at home compared to the hospital, perhaps because doctors and nurses who conducted births at facilities discouraged families from practising oil massage with mustard oil given its potentially harmful effects (14), and at the time, lack of information from randomized controlled trials demonstrating its benefits. Among families whose infants received emollient therapy in the hospital, about half perceived it to be superior to mustard oil and about half were unsure about what their future emollient-therapy practices would be, since they did not know which emollients would be available in the future. Further education of families, physicians, and nurses is needed to inform them about the potential benefits of oil massage when mustard oil is replaced with another oil, such as sunflower seed oil. Benefits may include improved skin condition and barrier function, resulting in reduced loss of transepidermal water and improved thermoregulation (5,7,10,14–16); absorption of fatty acids (17–21), contributing to improved nutrition; better somatic growth, neuro-development and infant-parent bonding (19–21,22–25); and improved skin integrity and reduced risk of nosocomial infection (13,14,26,27). If caregivers and health professionals conducting deliveries can be motivated to use inexpensive, readily-available oils, such as sunflower seed oil, that are beneficial to babies, this practice could have substantial public-health benefit.
